# The effects of circularly polarized light on mating behavior and gene expression in *Anomala corpulenta* (Coleoptera: Scarabaeidae)

**DOI:** 10.3389/fphys.2023.1172542

**Published:** 2023-03-31

**Authors:** Tong Li, Yueli Jiang, Xiaofan Yang, Huiling Li, Zhongjun Gong, Yifan Qin, Jing Zhang, Ruijie Lu, Guoshu Wei, Yuqing Wu, Chuantao Lu

**Affiliations:** ^1^ Henan Key Laboratory of Crop Pest Control, Key Laboratory of Integrated Pest Management on Crops in Southern Region of North China, Institute of Plant Protection, Henan Academy of Agricultural Sciences, Zhengzhou, China; ^2^ Plant Protection Institute, Hebei Academy of Agricultural and Forestry Sciences, Baoding, China; ^3^ College of Plant Protection, Hebei Agricultural University, Baoding, Hebei, China

**Keywords:** *Anomala corpulenta*, left circularly polarized light, mating behavior, scarab beetles, light stress

## Abstract

Light is an important abiotic factor affecting insect behavior. In nature, linearly polarized light is common, but circularly polarized light is rare. Left circularly polarized (LCP) light is selectively reflected by the exocuticle of most scarab beetles, including *Anomala corpulenta*. Despite our previous research showing that this visual signal probably mediates their mating behavior, the way in which it does so is not well elucidated. In this study, we investigated how LCP light affects not only mating behavior but also gene expression in this species using RNA-seq. The results indicated that disruption of LCP light reflection by females of *A. corpulenta* probably affects the process by which males of *A. corpulenta* search for mates. Furthermore, the RNA-seq results showed that genes of the environmental signaling pathways and also of several insect reproduction-related amino acid metabolic pathways were differentially expressed in groups exposed and not exposed to LCP light. This implies that *A. corpulenta* reproduction is probably regulated by LCP light-induced stress. Herein, the results show that LCP light is probably perceived by males of the species, further mediating their mating behavior. However, this hypothesis needs future verification with additional samples.

## Introduction

Vision is an important sense that triggers particular behaviors in most animals. In nature, polarized light is generated by atmospheric scattering of unpolarized sunlight and by sunlight reflecting off surfaces like water, leaves, and bodies (Wehner, 2001). Invertebrates, including insects (Horváth and Varjú. (1997); Dacke et al., 2002) and spiders (Dacke et al., 2001), can usually perceive linearly polarized light. However, only a small number of vertebrates are known to use polarization for object-based vision, which exclusively occurs in fish (Kamermans and Hawryshyn, 2011). Polarized light is known to mediate diverse behaviors in insects, including navigation (Labhart and Meyer, 2002), host recognition (Blake et al., 2019), and water avoidance (Shashar et al., 2005). Circularly polarized (CP) light is another form of polarized light that is less common in nature (Heinloth et al., 2018). Multiple studies have revealed that CP light can be perceived by mantis shrimp and that it affects their mating behavior and defensive behaviors (Chiou et al., 2008; Baar et al., 2014; Gagnon et al., 2015; Templin et al., 2017).

Michelson (1911) initially reported on reflection of polarized light in some scarab beetles (Michelson, 1911), with subsequent studies revealing that their exocuticle selectively reflects left circularly polarized (LCP) light (Finlayson et al., 2017; Bagge et al., 2020). The scarab beetle, *A. corpulenta* Motschulsky, is a destructive agricultural and horticultural pest in China. Its body has a metallic green color and LCP light reflects off its exocuticle. Our previous research showed that the mating behavior of *Anomala corpulenta* is probably regulated by a visual signal (Miao et al., 2015). However, the way in which LCP light affects the mating behavior of *A. corpulenta* has not been thoroughly investigated. In this study, we examine the effects of LCP light on *A. corpulenta* mating behavior. Furthermore, the effects of LCP light on their gene expression are revealed using RNA-seq, and the potential molecular responses to LCP light-induced stress are discussed.

## Materials and methods

### Insect collection

The *A. corpulenta* adults used in this study were trapped using black lamps in peanut fields in Yuanyang County, Henan Province (35.01 N, 113.69 E), in June 2022. The collected individuals were fed fresh peanut leaves and kept under room conditions of 25 °C with a 16:8 h light:dark regime.

### Mating experiments

To uncover the effects of LCP light on the mating behavior of *A. corpulenta*, we disrupted these effects by painting the *A. corpulenta* elytra with green nail enamel, which is similar to their body color. Three experimental groups were established: 1) a group in which only the males were painted, 2) a group in which only the females were painted, and 3) a group in which both males and females were painted. The behavior of these groups was compared in order to reveal the beetles’ specific sexual responses to LCP light during mating. A fourth group, consisting of normal (unpainted) *A. corpulenta*, was used as a control. The mating experiments were conducted as previously described (Miao et al., 2015), with a few modifications. First, 10 male and 10 female *A. corpulenta* beetles of one of the experimental groups were placed into a plastic glass pot and kept under natural conditions of <0.3 lx (night) and ∼700 lx (day) for 24 h. Five replicates were used for each condition. The number of mating pairs of *A. corpulenta* beetles was counted every half hour. Mating males and females were gently separated after being counted.

### Transcriptome sequencing of *Anomala corpulenta*

The effect of LCP light on gene expression in *A. corpulenta* was evaluated using transcriptome sequencing. First, adult beetles were placed into transparent plastic pots and initially kept under a daylight lamp for 3 h, then transferred into a dark room for 3 h. Thereafter, they were divided into an LCP light treatment group (exposed to LCP light at 600 lx for 3 h) and a control group (exposed to darkness (0 lx) for 3 h). A 50 W bromine tungsten lamp (OSRAM, Germany) was used as a light source with LCP light filtered using LCP film (Nitto Denko Corporation, Osaka, Japan). Five *A. corpulenta* pairs (i.e., five males and five females) were subjected to each treatment, and each condition was performed in triplicate. The heads of the individuals were then dissected and subjected to transcriptome sequencing.

Total RNA was extracted using TRIzol reagent (Invitrogen, CA, United States) following the manufacturer’s recommendations. The RNA integrity number (RIN) was obtained using the RNA 1000 Nano LabChip Kit on a Bioanalyzer 2100 (Agilent, CA, United States) to evaluate the quantity and purity of total RNA. Samples with RIN <7.0 were excluded from subsequent analyses. mRNA was enriched from ∼5 µg total RNA using poly-T oligo-attached magnetic beads with two rounds of purification. Thereafter, the purified mRNA was fragmented using divalent cations under high temperature. Subsequently, the cleaved RNA fragments were reverse-transcribed to create a final cDNA library as per the transcriptome sample preparation kit protocol (Illumina, San Diego, United States). Paired-end sequencing was performed on an Illumina NovaSeq™ 6000 platform. In this study, sequencing libraries were constructed and transcriptome sequencing was conducted by Lc-Bio Technologies Co., Ltd. (Hangzhou, China).

### Bioinformatics analyses

The adaptors of the row data were removed using Cutadapt (Version 1.9) (Martin, 2011), and low-quality bases were further trimmed using fqtrim (Version 0.94) (https://ccb.jhu.edu/software/fqtrim/). FastQC (Version 0.10.1) (http://www.bioinformatics.babraham.ac.uk/projects/fastqc/) was used to evaluate the quality of the cleaned data, with low-quality data eliminated for subsequent analyses. *De novo* transcriptome assembly was performed using Trinity (Version 2.4.0) (Grabherr et al., 2011). The longest assembled transcript of a given gene was defined as a unigene. The functions of unigenes were predicted using DIAMOND (Version 0.7.12) (Buchfink et al., 2015) against the non-redundant (Nr) protein sequence database and the Kyoto Encyclopedia of Genes and Genomes (KEGG). An e-value threshold of 1 × 10^−5^ was used in the searches. GO (Gene Ontology) annotations were mapped to the GO terms in the Gene Ontology database (http://www.geneontology.org/) using Blast2GO (Version 2.3.5) (Conesa et al., 2005). Transcripts per million (TPM) of unigenes were calculated using Salmon (Version 0.8.2) (Patro et al., 2017) to evaluate their expression levels. The edgeR R package (Robinson et al., 2010) was used to help filter differentially expressed genes (DEGs) with a threshold of absolute log2 (FC, fold change) ≥1 and statistical significance (*p*-value) < 0.05. Principal component analysis (PCA) of the samples was carried out using the gene expression matrix and subsequently visualized using the stats R package.

GO and KEGG enrichment was performed for the DEGs and visualized using the clusterProfiler R package (Yu et al., 2012). The gene–concept network highlighted the interactions between DEGs, with significantly enriched KEGG pathways further constructed using clusterProfiler. The expression patterns of the DEGs among the individuals were visualized using heat maps constructed using TBtools (Chen et al., 2020). The DEGs were further verified by real-time quantitative reverse transcription PCR (RT-qPCR). First, cDNA was synthesized using the First-Strand cDNA Synthesis Kit (Toyobo, Shanghai); RT-qPCR was then performed using the SYBR Green Real-Time PCR Master Mix Kit (Toyobo) on a Mastercycler® ep realplex system (Eppendorf). The relative expression of DEGs was normalized to the *A. corpulenta* β-actin gene as previously described (Livak and Schmittgen, 2001; Bustin et al., 2009). All RT-qPCR assays were conducted with three biological replicates and further analyzed via one-way analysis of variance (ANOVA). The primers used in this study were listed in Supplementary Table S1.

## Results

### Change in the body color of *Anomala corpulenta* under circularly polarized light

**FIGURE 1:**
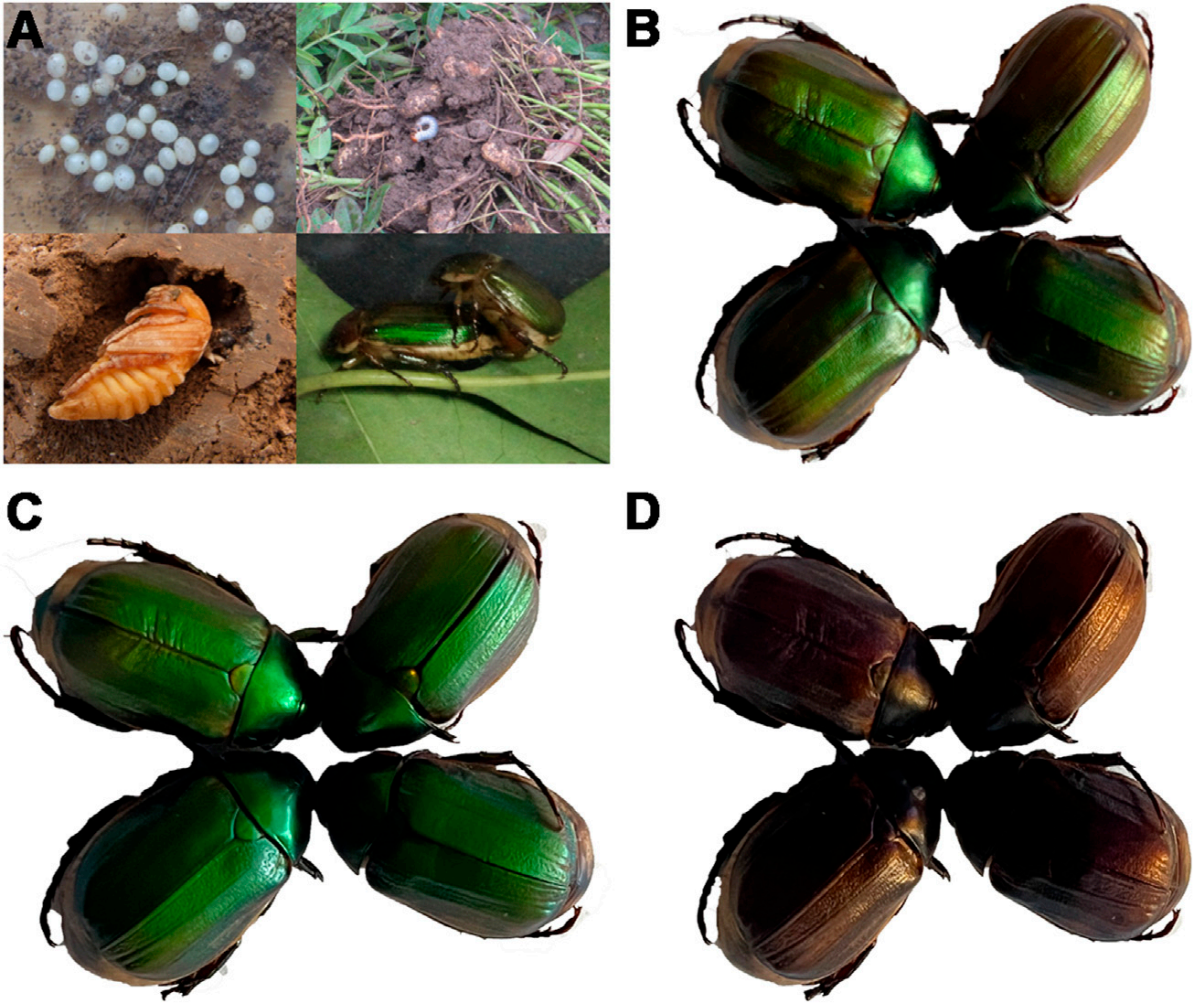
Developmental stages of *Anomala corpulenta* and photographs of the beetles under different polarizing films. **(A)** Developmental stages of *A. corpulenta*. **(B)** Photograph showing appearance without polarizing film. **(C)** Photograph showing appearance with left circularly polarizing film. **(D)** Photograph showing appearance with right circularly polarizing film.

In Henan Province, *A. corpulenta* is one of the major pests of peanut fields (Figure 1A). Under normal light conditions, their body color is green with a little brown (Figure 1B). However, this color is clearly altered by circularly polarized light. In particular, the *A. corpulenta* cuticle selectively reflects LCP light (Figure 1C) rather than right circularly polarized (RCP) light (Figure 1D).

### Effects of LCP light on the mating behavior of *Anomala corpulenta*

**FIGURE 2:**
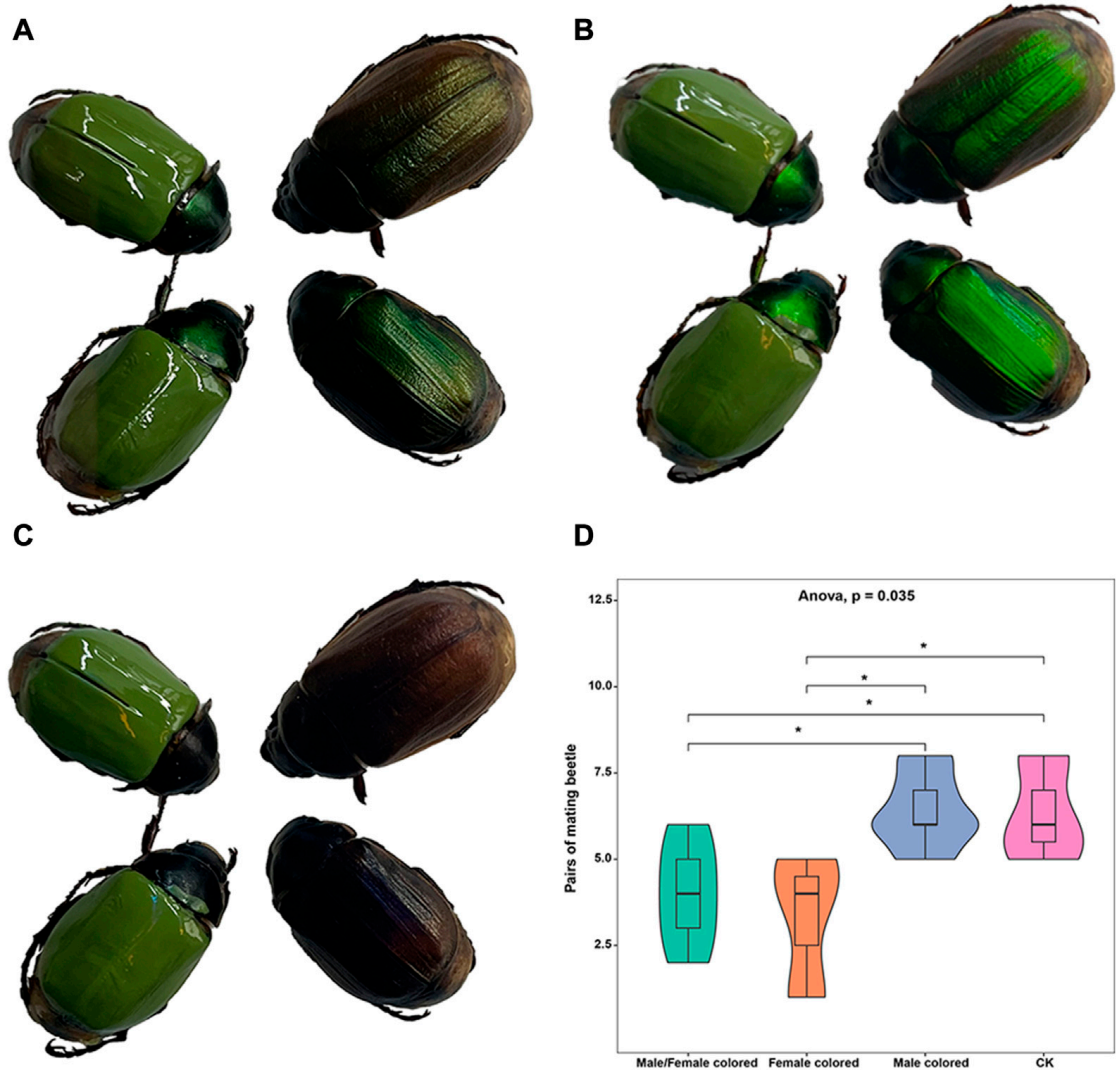
Photographs of painted beetles under different polarizing films and results of mating experiment. **(A)** Photograph showing appearance without polarizing film. **(B)** Photograph showing appearance with left circularly polarizing film. **(C)** Photograph showing appearance with right circularly polarizing film. **(D)** Results of the mating experiment with painted and unpainted members of *A. corpulenta*.

In this study, to eliminate the effect of body color and evaluate the potential effects of LCP light on the mating behavior of *A. corpulenta*, we colored the elytra of some individuals with green nail enamel (Figure 2A). The results showed that the reflection of circularly polarized light by *A. corpulenta* was greatly disrupted by painting of the elytron (Figures 2B, C). Furthermore, the number of pairs of mating beetles differed significantly between the groups with and without elytron-painting treatment (ANOVA: F = 4.875, *p* = 0.035) (Figure 2D). Multiple comparisons indicated that, relative to the control group, the number of pairs of mating beetles was significantly reduced in the group with painted females (LSD test: *p* = 0.012) but there was no significant difference in the case of the group with painted males (LSD test: *p* = 0.739).

### Analyses of the *Anomala corpulenta* transcriptome

**FIGURE 3:**
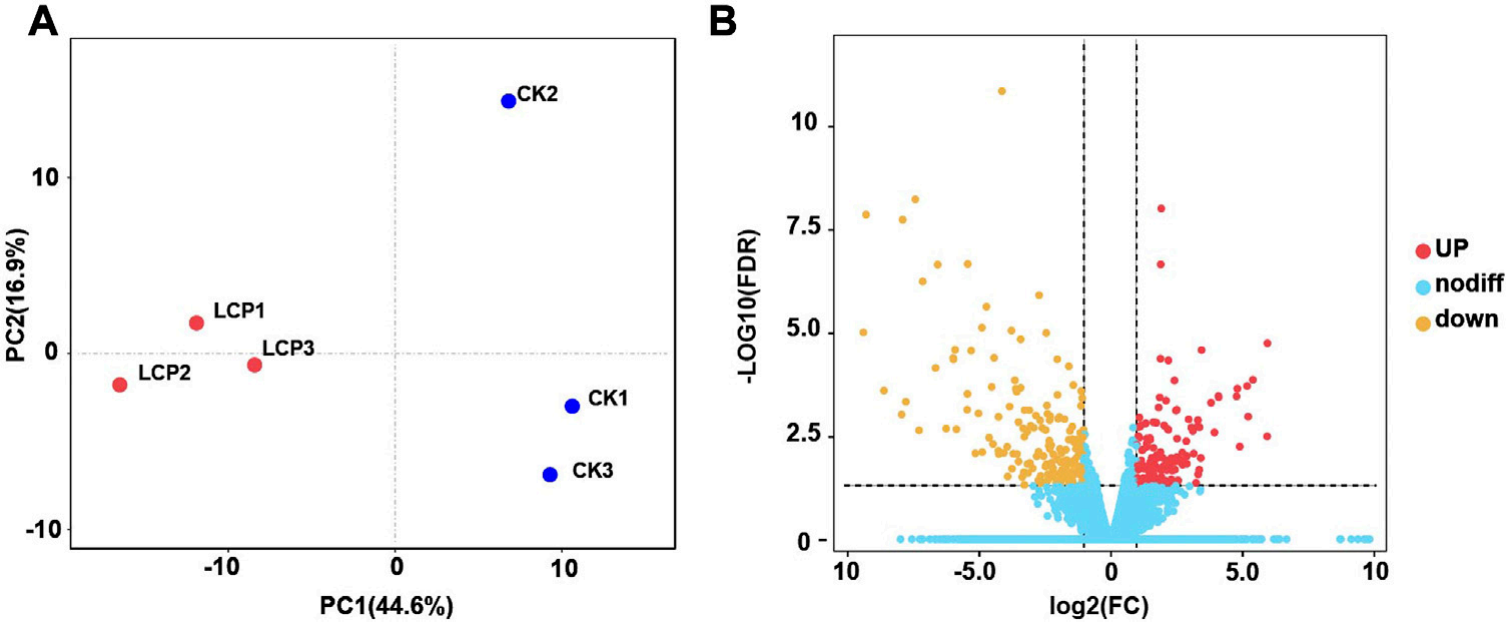
Principal component analysis (PCA) and gene expression of *Anomala corpulenta* subjected to left circularly polarized light. **(A)** PCA using the gene expression matrix. **(B)** Volcano plot of gene expression.

In this study, we identified 32,619 unigenes with 37.13% GC content assembled in the *A. corpulenta* transcriptome. The N50 of these unigenes was 1,578 bp. We predicted the functions of 15,786 and 10,619 unigenes against the Nr and KEGG databases, respectively. Furthermore, we annotated 11,133 unigenes in the GO predictions. The PCA showed that the LCP light treatment and control groups were well distinguished (Figure 3A). Furthermore, 315 unigenes were filtered as DEGs, with 176 and 139 being significantly downregulated and upregulated, respectively, at the filtered thresholds (Figure 3B). We also observed similar expression patterns of the representative DEGs *via* RT-qPCR (Supplementary Figure S1), which further confirmed the RNA-seq results.

### Predictions of the functions of DEGs

**FIGURE 4:**
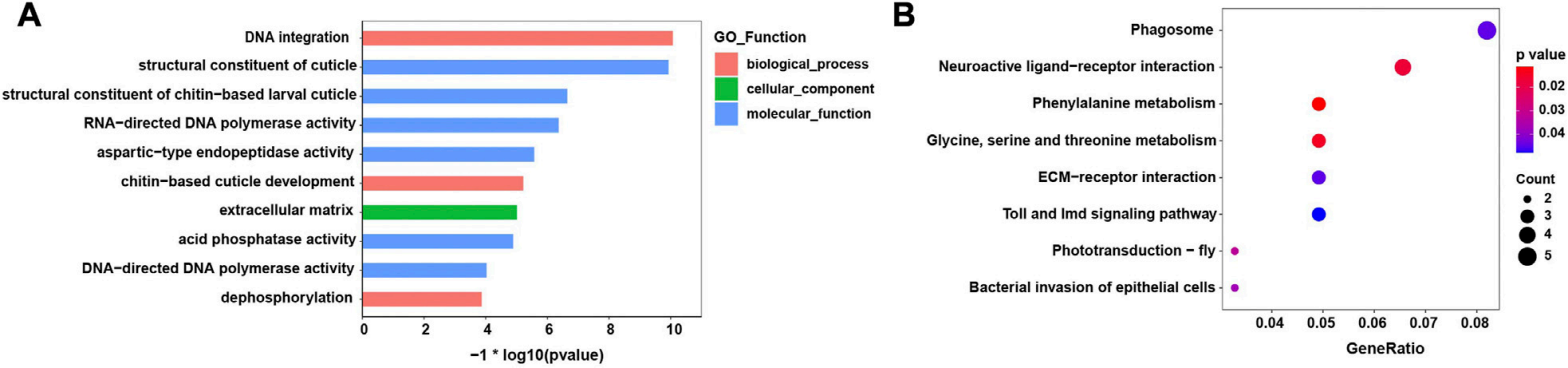
Enriched GO terms and KEGG pathways of the differentially expressed genes (DEGs). **(A)** Top ten GO significantly enriched terms (FDR< 0.05) **(B)** KEGG significantly enriched pathways (FDR< 0.05).

The top 10 enriched GO terms and KEGG pathways are shown in Figure 4. In the GO analysis, most enriched GO terms were clustered under molecular functions. Moreover, the enriched GO terms mainly related to the process of integration and synthesis of DNA, while terms relating to contributions to the structural integrity of the insect cuticle also appeared (Figure 4A). In the KEGG analysis, the significantly enriched pathways were those falling under amino acid metabolism and insect phototransduction, such as glycine, serine, and threonine metabolism and phototransduction–fly (Figure 4B).

**FIGURE 5:**
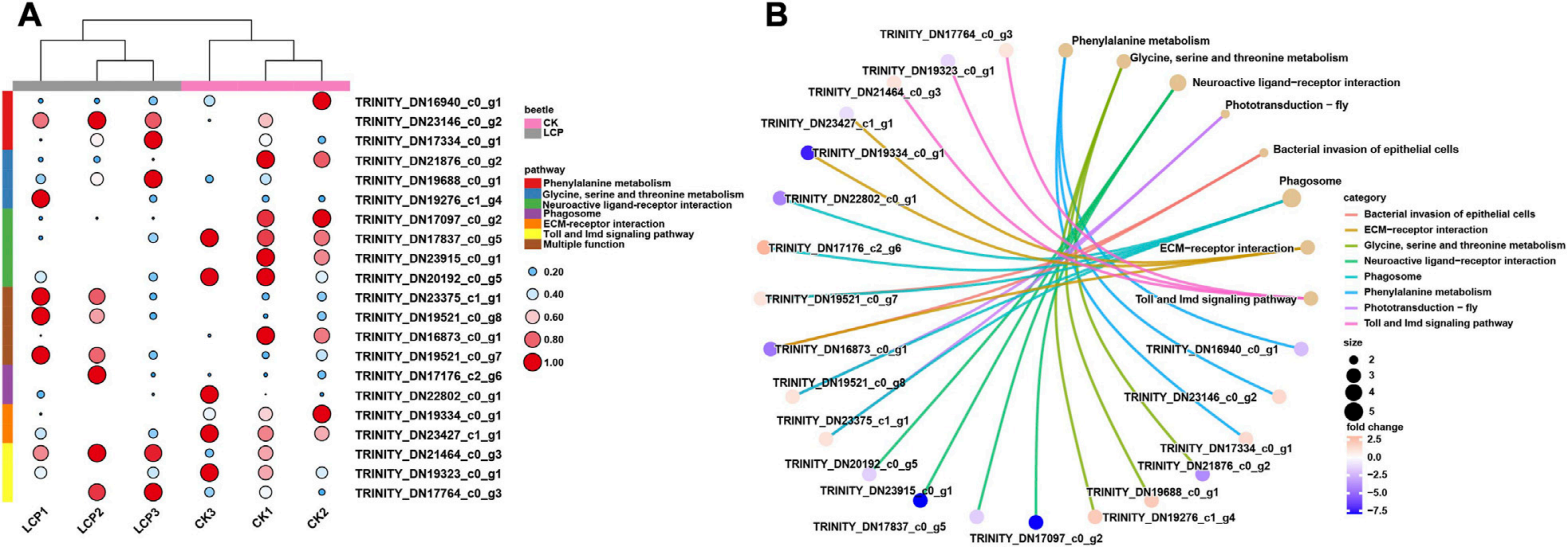
Expression patterns and gene–concept network of DEGs. **(A)** Expression patterns of DEGs that related to the significantly enriched pathways. Log2 TPM values are further scaled by row using the zero-to-one method and then indicated by the color and area of the circles. **(B)** Gene–concept network of DEGs among significantly enriched pathways.

Heat maps were also used to visualize differences between the treatment groups in the expression patterns of DEGs in the significantly enriched pathways (Figure 5A). The results showed that the patterns of DEG expression generally clustered by treatment group, and expression patterns varied between groups within the same enriched pathway. In particular, DEGs enriched in the neuroactive ligand–receptor interaction were significantly downregulated in the LCP light group, whereas those enriched in phototransduction were significantly upregulated.

Finally, the interactions between DEGs and significantly enriched pathways are presented in the gene-concept network (Figure 5B). The results show that four DEGs were enriched in multiple pathways. Within these pathways, two DEGs were shared between the phototransduction–fly and phagosome pathways, one DEG was shared between the bacterial invasion of epithelial cells and ECM–receptor interaction pathways, and one DEG was shared between the bacterial invasion of epithelial cells and phagosome pathways.

## Discussion

In nature, polarized light is generated by atmospheric scattering of unpolarized sunlight and its reflection off surfaces like water, leaves, and bodies (Wehner, 2001). Polarized light of the form commonly referred to as linearly polarized light plays a role in insect navigation (Wehner, 1976; Krapp, 2007), assists in the predation behaviors of cuttlefish and horseflies (Shashar et al., 1998; Shashar et al., 2000; Meglic et al., 2019), and affects recognition of host and oviposition sites in dragonflies and butterflies, respectively (Wildermuth, 1998; Blake et al., 2019). Circularly polarized light is another form of polarized light that is less common in nature (Heinloth et al., 2018). Previous studies have indicated that circularly polarized light is a covert signal in the intraspecific communication of stomatopod crustaceans (Templin et al., 2017), and that it can alter their mating selections (Chiou et al., 2011; Baar et al., 2014).

Left circularly polarized (LCP) light is selectively reflected by the exocuticle of scarab beetles (Coleoptera: Scarabaeidae) (Finlayson et al., 2017; Bagge et al., 2020). However, its role in beetles has not been thoroughly investigated. Although our previous research showed that the mating behavior of *A. corpulenta* is affected by visual signals, the roles of body color and LCP light in the mate choices of *A. corpulenta* are not well understood (Miao et al., 2015). To investigate how LCP light affects the mating behavior of *A. corpulenta*, we disrupted the reflection of LCP light by members of this species, while retaining their general green body color, by painting their elytra with green nail enamel. The results indicated that, compared with controls, the number of mating pairs of *A. corpulenta* was significantly reduced when LCP light reflection was disrupted in female *A. corpulenta*, but not in male *A. corpulenta*. In beetles, male insects do most of the searching for mates (Muniz et al., 2018). Thus, this finding implies that LCP light reflected by the exocuticle of females of *A. corpulenta* is probably perceived by males of the species, which improves their ability to find mates, as previously reported in *Heliconius* butterflies (Sweeney et al., 2003). However, the circularly polarized light-detecting capability of scarab beetles is debatable. While the jewel scarab, *Chrysina gloriosa*, can distinguish circularly polarized light, another jewel scarab, *C. woodi*, exhibits no phototactic discrimination between linear and circularly polarized light (Brady and Cummings, 2010). Furthermore, in another study, four scarab beetles showed no behavioral response under circularly polarized light treatments (Blaho et al., 2012). Hence, despite the demonstration in this study of potential interactions between LCP light and mating behavior in *A. corpulenta*, firm conclusions cannot be drawn on their LCP light-perceiving capacity. Therefore, further behavioral experiments with more samples are required.

Previous studies indicate that light stress strongly alters the gene expression patterns of insects (Yang et al., 2021; Wang et al., 2023). In this study, we investigated the effect of LCP light on the gene expression patterns of *A. corpulenta*. The results showed that the insect phototransduction pathway was enriched. Phototransduction is a well-known signaling cascade that converts light energy into an electrical signal. In insects, the expression of phototransduction-related genes is significantly modified by exposure to different light environments (Macias-Munoz et al., 2019) and even viral infections (Bhattarai et al., 2018b). Another environmental information signaling pathway, including neuroactive ligand–receptor interaction, was also enriched. Although the neuroactive ligand–receptor interaction pathway is known for responding to diverse environmental stresses (Cao et al., 2013; Yadav et al., 2017; Bhattarai et al., 2018a), it has also been found to be vital in adaptations in invasive beetles (Liu et al., 2021). Furthermore, some amino acid metabolic pathways that regulate insect reproduction were also found to be significantly enriched. Recent studies indicate that phenylalanine metabolism regulates reproduction and mating behavior in flies and mosquitos (Proline et al., 2014; Arya et al., 2021), while glycine, serine, and threonine metabolism are involved in sex pheromone biosynthesis in the oriental fruit fly (Gui et al., 2023). Overall, when *A. corpulenta* beetles were subjected to LCP light stress, not only was the expression of insect environmental signaling pathway-related genes affected, but that of amino acid metabolism pathways related to insect reproduction was also affected. This indicates that LCP light probably regulates reproduction in *A. corpulenta*.

In conclusion, we investigated the effect of LCP light on the mating behavior of *A. corpulenta* and also examined how exposure to this affects gene expression in this species. The results indicated that LCP light probably affects the mate-searching capabilities of male *A. corpulenta* beetles, with multiple insect environmental signaling and reproduction-related pathways being significantly enriched when they are subjected to LCP light-induced stress.

## Data Availability

The datasets presented in this study can be found in online repositories. The names of the repository/repositories and accession number(s) can be found at: NCBI, accession ID: PRJNA907534.

## Supplementary material

SUPPLEMENTARY FIGURE S1 Verification of DEG expression via RT-qPCR.
