# Supplementary figures and images for: The effects of circularly polarized light on mating behavior and gene expression in Anomala corpulenta (Coleoptera: Scarabaeidae)
Source: Front Physiol. 2023 Mar 31;14:1172542. doi: 10.3389/fphys.2023.1172542 (PMC10102372; doi:10.3389/fphys.2023.1172542)

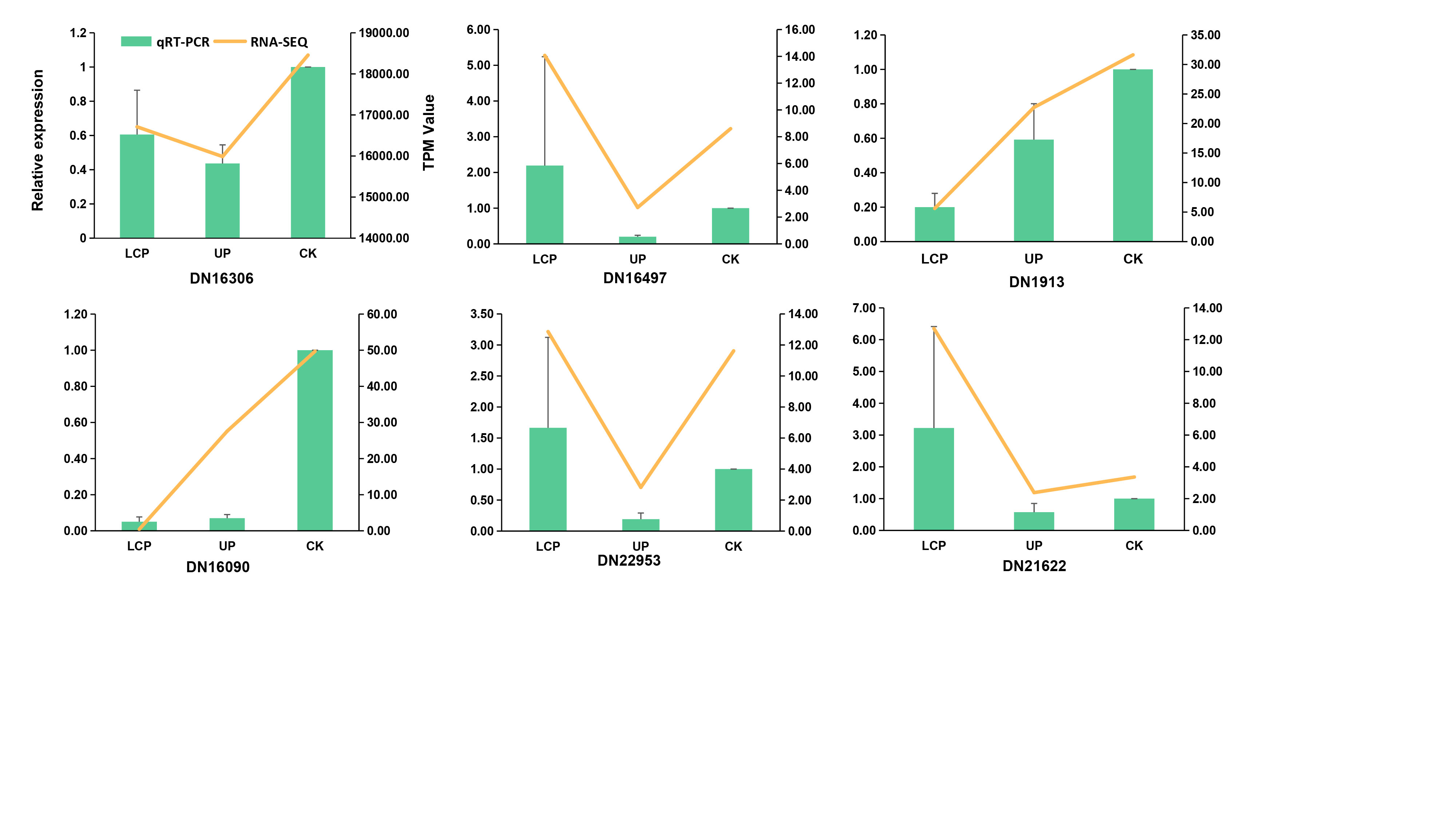

Supplement: Supplementary file 1 [file Image1.JPEG]
